# Point-of-Care Ultrasonography (POCUS) in obstetric anesthesia fellowship training: survey of North American programs

**DOI:** 10.1016/j.bjane.2024.844547

**Published:** 2024-08-03

**Authors:** Juliana Barrera Ramirez, Xao Xu Chen, Nathan Ludwig, Indu Sudha Singh, Ilana Sebbag

**Affiliations:** a Western University, Schulich School of Medicine & Dentistry, Department of Anesthesiology & Perioperative Medicine, London, Canada; b BC Women's Hospital and Health Centre, Department of Anesthesia, Vancouver, Canada; c McMaster University, Faculty of Health Sciences, Department of Medicine, Hamilton, Canada

Dear Editor,

Point-of-Care Ultrasound (POCUS) has become an essential tool in bedside assessment and management of critically ill patients since its introduction to the clinical setting. Its portability, safety, and noninvasive nature have facilitated its use in various areas, including obstetric anesthesia.^1^ While POCUS training has been successfully integrated into the teaching curriculum of other specialties,^2^ implementation of a similar curriculum in anesthesia residency programs remains highly variable.^3^ The current state of POCUS education in obstetric anesthesia fellowships across North America remains unclear. This survey aimed to explore the prevalence, characteristics, current applications, and potential barriers of POCUS education in these programs.^2^

Following approval by the Health Sciences Research Ethics Board at Western University, (HSREB #115106) a 12-question web-based survey was sent to a total of 42 obstetric anesthesia fellowship program directors from June 2021 to August 2021. Contact information was obtained from the official listing of fellowship programs on the Society for Obstetric Anesthesia and Perinatology (SOAP) website. This included both Canadian and American institutions. One institution was excluded from the survey due to inaccurate contact information. Only one survey was allowed per program and all answers were collected anonymously through the Qualtrics online survey platform (https://www.qualtrics.com). Survey content was adapted from previous literature on ultrasonography in anesthesia residency program education.^3^^,^^4^ All participants provided informed consent prior to completing the survey.

Twenty-one (50%) fellowship program directors answered the survey, of which 18 (86%) were programs located in the United States and 3 (14%) in Canada. The main areas for POCUS utilization included vascular access (91%), neuraxial blocks (81%), abdominal blocks (71%), transthoracic echocardiography (71%), lung (43%), gastric (38%), IVC (38%), and airway assessment (9.5%). Program directors perceived that obstetric anesthesia fellows’ competency in POCUS was extremely important (60%), very important (20%), moderate (15%), and slightly important (5%). The main modality identified for delivery of POCUS teaching was informal teaching (33%), followed by formal longitudinal programs (29%), elective rotations (14%), and mandatory POCUS rotations (9.5%). Out of the participating programs, 70% had dedicated staff as POCUS educators while 30% did not. Only 9.5% of programs had mandatory POCUS rotations as part of their curriculum.

According to our findings, current POCUS training is delivered using a variety of teaching modalities. In fact, 11 out of 21 programs use e-learning modules and 8 out of 21 provide structured lectures. Moreover, most POCUS teaching takes place in the form of informal bedside teaching. These findings are in keeping with previous literature^3^^,^^5^ and are in line with the 2021 Canadian Guidelines.^5^ Varying teaching modalities including lectures, e-learning modules, and hands-on experience with the use of a portfolio-building approach has been previously recommended.^5^ Therefore, integrating these learning modalities within a cohesive POCUS training curriculum is important to ensure that trainees obtain a minimum level of competency and experience upon completion of their fellowship.

This survey highlights the lack of structured POCUS curricula in obstetric anesthesia fellowship programs. This may be attributed to several barriers reported by fellowship directors, including number of trained faculty, available time during clinical care, availability of equipment, interest of learners, and costs. The recent consensus of Canadian anesthesiology academic centers recommended the use of an “offline approach” to overcome the shortage of POCUS trained faculty.^5^ This strategy enables the trainee to digitally store videos and still images that could be reviewed by an off-site expert. Furthermore, adoption of structured POCUS curricula would ensure that future obstetric anesthesia consultants have a minimum level of competency in POCUS, thereby, mitigating the current lack of trained faculty.

The more recent integration of handheld POCUS devices into clinical practice provides a more affordable alternative to standard ultrasound consoles.^6^ In addition, they are highly portable, which facilitates their frequent use in busy clinical settings.^6^ These devices are a plausible option to overcome barriers of equipment availability and costs, as teaching centers may be able to afford multiple handheld devices instead of a single more expensive ultrasound console. Furthermore, their portability will lead to less disruption to clinical workflow and facilitate bedside teaching.

This study has several limitations inherent to surveys. Despite our efforts to maximize responses, our survey had a response rate of 50%. A well-established contact information database was implemented; however, this did not translate into a higher response rate. This may be in part due to the survey being distributed during the summer months when more clinicians are likely to be absent. Furthermore, obstetric anesthesia fellowship programs in the United States were over-represented in our sample with only 14% (3/21) of respondents being from Canada. Given that we used a single contact information database, we were limited by the programs registered within SOAP. In fact, not all Canadian obstetric anesthesia fellowship programs are part of this database, and therefore, we had an inherent response bias. A previous survey by Dominguez and colleagues had a response rate of 59% out of which only 14% represented Canadian fellowship programs,^4^ which is very similar to our results. While our survey represents a similar geographic distribution to previous studies, we cannot generalize our findings to all Canadian programs. Future surveys evaluating POCUS utilization in Canadian obstetric anesthesia fellowships are needed to better delineate current practice and barriers specific to Canada.

In conclusion, this survey highlights the need for a well-structured POCUS curriculum in obstetric anesthesia fellowship programs. This will ensure that all trainees receive uniform and adequate training in POCUS, which is a critical skill for obstetric anesthesiologists. The main barriers to implementing such a curriculum are the lack of trained faculty, time, equipment availability, and costs.

## Conflicts of interest

The authors declare no conflicts of interest.
